# The right bug in the right place: opportunities for bacterial vaginosis treatment

**DOI:** 10.1038/s41522-022-00295-y

**Published:** 2022-05-02

**Authors:** Shengru Wu, Luisa Warchavchik Hugerth, Ina Schuppe-Koistinen, Juan Du

**Affiliations:** 1 Centre for Translational Microbiome Research (CTMR), Department of Microbiology, Tumor, and Cell Biology, Karolinska Institutet, Stockholm, Sweden; 2 Science for Life Laboratory, Stockholm, Sweden

**Keywords:** Clinical microbiology, Antimicrobials

## Abstract

Bacterial vaginosis (BV) is a condition in which the vaginal microbiome presents an overgrowth of obligate and facultative anaerobes, which disturbs the vaginal microbiome balance. BV is a common and recurring vaginal infection among women of reproductive age and is associated with adverse health outcomes and a decreased quality of life. The current recommended first-line treatment for BV is antibiotics, despite the high recurrence rate. Live biopharmaceutical products/probiotics and vaginal microbiome transplantation (VMT) have also been tested in clinical trials for BV. In this review, we discuss the advantages and challenges of current BV treatments and interventions. Furthermore, we provide our understanding of why current clinical trials with probiotics have had mixed results, which is mainly due to not administering the correct bacteria to the correct body site. Here, we propose a great opportunity for large clinical trials with probiotic strains isolated from the vaginal tract (e.g., *Lactobacillus crispatus*) and administered directly into the vagina after pretreatment.

## Introduction

The vaginal microbiome is commonly dominated by one species of *Lactobacillus* (e.g., *L. crispatus, L. gasseri, L. iners*, or *L. jensenii*)^1–3^. Some women have a vaginal microbiome that is dominated by non-*Lactobacillus* species, especially Black and Hispanic women^4^. Bacterial vaginosis (BV) is a condition in which the vaginal microbiome has a deficiency of lactic acid-producing bacteria with increased numbers of anaerobic bacteria such as *Gardnerella*, *Atopobium*, *Megasphera*, *Prevotella*, and *Sneathia*^1–3^. Common BV symptoms include vaginal discharge, increased vaginal pH, itching, fish-like odor, and burning when urinating^5^. Given the high percentage of women with low vaginal *Lactobacillus spp*. abundance but lacking BV symptoms, whether these women are healthy or have asymptomatic BV has remained a subject of debate^1^.

BV prevalence varies geographically and ethnically, and can affect >50% of women in some countries^6^. BV is diagnosed using Amsel’s criteria or Nugent score, with Amsel’s criteria more commonly used in the clinic^5^. Amsel’s criteria combines inspection of vaginal secretions, pH measurement, visual inspection under microscopy, and the Whiff test, whereas the Nugent score focuses exclusively on scoring Gram-stained microscopy images. The BV definition based on DNA sequencing of vaginal secretions is referred to as molecular BV^7,8^.

**Fig. 1:**
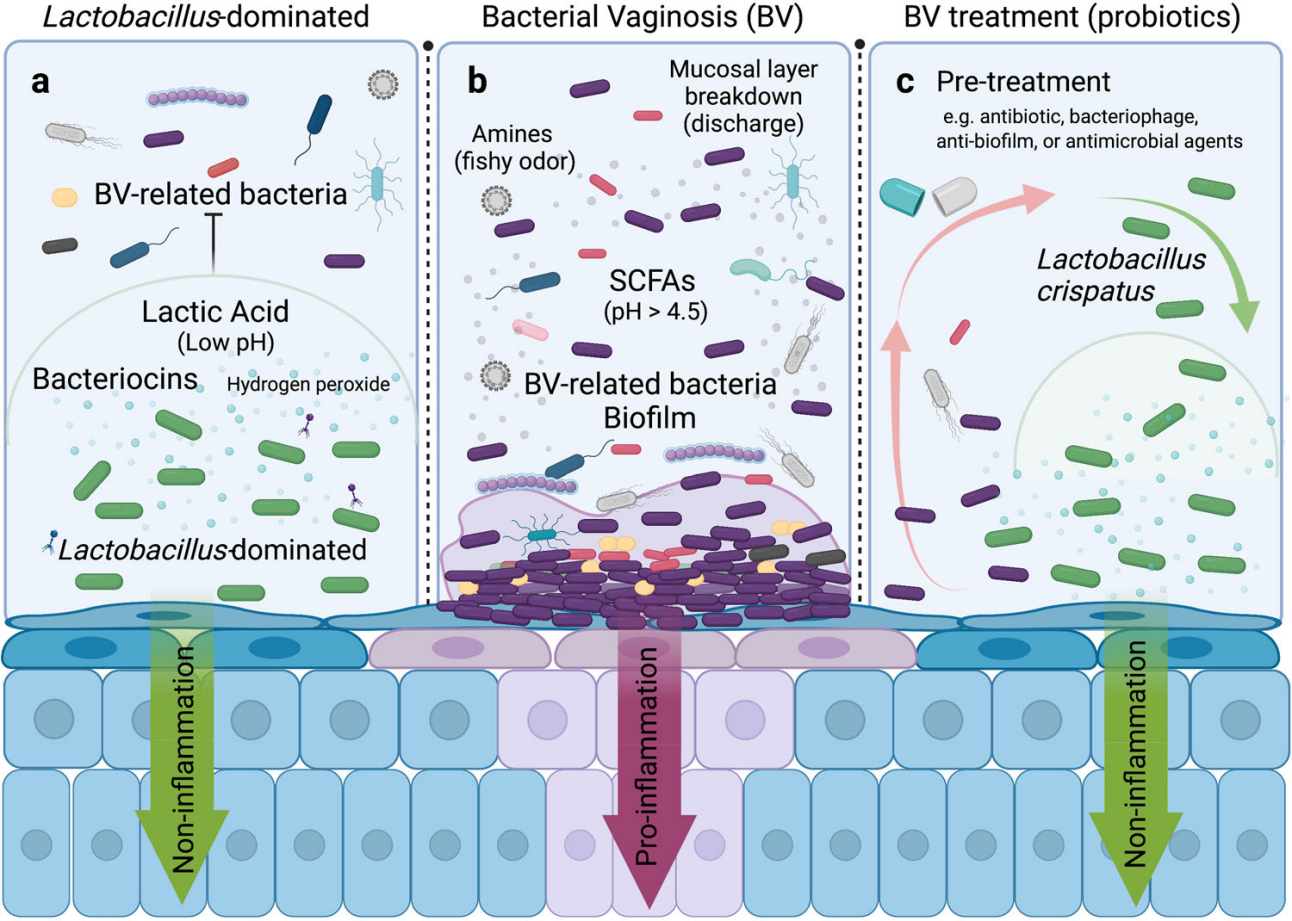
Overview of the strategy on vaginosis treatment with probiotics. **a** *Lactobacillus*-dominated vaginal microbiome environment. Vaginal *Lactobacillus* species, such as *Lactobacillus crispatus*, produce lactic acid, bacteriocins, and hydrogen peroxide (H_2_O_2_), which may provide protection against bacterial vaginosis (BV) related bacteria and other infections. **b** BV microbiome environment. BV-related bacteria (mainly *Gardnerella*) induce inflammation in the vaginal tract and form a biofilm on vaginal epithelial cells. The latter probably increases antibiotic resistance and refractoriness to probiotic treatment. Short chain fatty acids (SCFAs) produced by BV-related bacteria, increase vaginal pH. In addition, catabolism of amino acids and mucosal proteins results in amines and a thinner mucosal layer in the vaginal tract. **c** Treatment of BV with probiotics. Pretreatment such as antibiotic, bacteriophage, anti-biofilm, or antimicrobial agents, in combination with vaginal probiotic species and vaginal administration, increase the probability of successful colonization. Note: figure was created with BioRender.com.

Recent studies have provided insights into the relationship between the vaginal microbiome environment and BV symptoms. In the *Lactobacillus*-dominated vaginal microbiome, various antimicrobial substances are produced, including lactic acid, bacteriocins, and hydrogen peroxide (H_2_O_2_), which play essential roles in protecting against potential pathogens^9–11^. Vaginal fluids are rich in glycogen, which is broken down into simpler carbohydrates by human alpha-amylase^12,13^. *Lactobacillus* species metabolize these carbohydrates, producing lactic acid and maintaining an acidic environment^9,14^. Bacteriocins, such as bacteriocins IIa, IIc, J46, acidocin lF221A, gassericin T, and type-A lantibiotic, produced by *Lactobacillus* species exhibit bactericidal activity^10^. Although H_2_O_2_ level has been linked to a healthy vaginal environment, its role in vaginal microbiome protection is still under investigation^15,16^. Furthermore, cervicovaginal secretions from women with *L. crispatus*-dominated vaginal microbiome show lower levels of genital inflammatory scores^17,18^ (Fig. 1). By contrast, vaginal fluids in BV are characterized by higher concentrations of short chain fatty acids (SCFAs), such as acetate, propionate, butyrate, and succinate, with vaginal pH elevated over 4.5^19,20^. Also, catabolism of amino acids results in amines that are responsible for the fishy odor, and catabolism of mucosal proteins results in a thinner mucosal layer and the production of a thin homogenous discharge^21^. Elevated cytokine and chemokine levels in the vaginal tract have also been observed in women with BV^22^ (Fig. 1).

The standard of care treatment for BV is antibiotics. Live biopharmaceutical products, defined by the United States Food and Drug Administration (FDA) and the European Pharmacopeia as “a biological product that contains live organisms; is applicable to the prevention, treatment, or cure of a disease or condition of human beings; and is not a vaccine”, or generally called “probiotics,” defined as “live microorganisms that, when administered in adequate amounts, confer a health benefit on the host” by the Food and Agriculture Organization of the United Nations and the World Health Organization and revised by the International Scientific Association for Probiotics and Prebiotics, have been evaluated as BV treatments in clinical trials for decades with mixed results. Recently, vaginal microbiome transplantation (VMT), the process of transferring the microbiome of a healthy donor to an individual as a therapeutic alternative, has been tested to restore the vaginal microbiome. In this review, we discuss the advantages and disadvantages of these interventions and provide our considerations of what needs to be contemplated for future clinical trials with probiotics.

## Advantages and limitations of current methods used for treating BV

### Antibiotics

Antibiotics are widely used for BV treatment and have an effective initial cure rate varying between 80% and 90% 1 month after treatment^1,23,24^. The recommended antibiotics for BV and recurrent BV are metronidazole and clindamycin in the clinic^1,25^, which can be administered orally or intravaginally^1,24^. These recommended regimens have similar efficacy for BV treatment, with minor differences in recurrence rates^26,27^. Other tested antibiotics include tinidazole and secnidazole, which have similar activity in vitro against a range of microorganisms associated with BV^28^.

Recurrent BV is a common problem associated with the treatment of BV and presents as repeated cases of BV after the initial cessation of symptoms. Available research suggests that recurrent BV may be caused by a relapse of infection due to an inability to reestablish a *Lactobacillus*-dominated vaginal microbiome or the ineffective suppression of BV-related bacteria^1^. Recurrence of BV can also indicate persistent BV, where a positive BV diagnosis remains unchanged after treatment^29,30^. A high rate of BV recurrence after 1 year has been observed, ranging from 50% to 100% depending on the antibiotic used and geographic locations, underscoring the need for additional treatments^1,23,30^. Other factors that could affect the vaginal microbiome and potentially the efficiency of BV treatment include age (before puberty or after menopause), pregnancy, sexual intercourse, and other diseases or medical conditions^31,32^.

The advantages of treatment with antibiotics are their availability and convenience for clinical use. Patients can easily administer antibiotics at home with instruction. Also, since BV is characterized by the overgrowth of anaerobic bacteria, reduced vaginal bacterial load following antibiotic treatment may provide the chance for *Lactobacillus* species to compete for nutrients and biological niches again. Overall, a large proportion of women with BV have been cured after one-time treatment in a short period of time^27,33,34^.

However, the main issue with using antibiotic treatment for BV is the high rate of recurrence within months of treatment^23^. Relapse may occur when vaginosis-related bacteria re-colonize and take over the vaginal microbiome. Furthermore, vaginosis-related bacteria that recur after antibiotic treatment, such as *Gardnerella vaginalis* and *Atopobium vaginae*, may have higher resistance and become less sensitive to another round of antibiotic treatment^29,30,35^. Also, orally administered metronidazole and clindamycin disturb the healthy gut microbiome^36,37^, whereas even local usage of antibiotics is a risk factor for vulvovaginal candidiasis^38,39^.

Thus, it is important to follow the dynamics of the vaginal microbiome for at least 6 months following treatment to assess complete clinical cure endpoints^23^. Moreover, restoring the vaginal microbiome after antibiotic treatment (e.g., with probiotics or VMT) will assist the recovery of the vaginal environment and complete the whole treatment procedure (Fig. 1). Therefore, evaluation of additional methods for BV treatment and prevention, especially recurrent BV, will be of great value in the clinic.

### Probiotics

With sequencing information obtained by the human microbiome project and many other vaginal microbiome investigations, we have learned that a large proportion of women without gynecological symptoms have a vaginal microbiome dominated by either *L. crispatus* or *L. iners*^2–4^. While an *L. crispatus*-dominated vaginal microbiome is related to a healthy vaginal condition, *L. iners*-dominated and specially non-*Lactobacillus*-dominated vaginal microbiomes are linked to a higher risk for infections, such as human papillomavirus and *Chlamydia trachomatis*^1,2^.

Using a single strain or mixtures of *Lactobacillus* strains, especially vaginal *L. crispatus*, could have many benefits. It avoids the risk of introducing other vaginal bacteria related to vaginosis or infections, such as *G. vaginalis* and *Prevotella* species. Once the *Lactobacillus* strains colonize, the produced lactic acid and bacteriocins could lower vaginal pH, inhibit BV-related pathogens, and potentially prevent BV recurrence in the long term^9–11,14^. It is easier to culture single bacterial strains in large batches under controlled standard conditions. It is also cheaper for clinical use and prevents the possibility of transferring cells and untested organisms from donor women, as in VMT.

On the other hand, probiotics only contain bacterial strains without other potential beneficial factors, such as bacteriophages or molecules that assist in the growth and colonization of *Lactobacillus* species. The colonization by *Lactobacillus* strains could be influenced by many factors including resident vaginal bacteria, glycogen, and lactic acid concentration, sexual intercourse, hormonal changes, and bleeding^40–42^. Moreover, the main concern for a single *Lactobacillus* strain is whether one strain would fit all genetic and immunological backgrounds, given a large variation of *L. crispatus* genomes in the vaginal microbiome^43,44^. If a mixture of strains should apply, it is still unclear which strains should be selected, and whether all mixtures will fit and colonize in women regardless of the geographic and ethnic background. If different *Lactobacillus* strains compete in the vaginal tract and further hinder their colonization or function, more strains would not necessarily have a stronger effect than a single strain. Finally, when mixed strains are used, each *Lactobacillus* strain needs to pass the standard of federal agency, which is a larger challenge than a single strain. Like VMT, the use of probiotics also lacks uniform and effective policy supervision. The lack of a standardized manufacturing process focused on the effectiveness and safety of probiotics, including the proper species, dose, administration method, presence of contaminating microorganisms, and auxiliary ingredients of probiotics, adds to uncertainty around the results for probiotics^45^.

### Potential factors that influence the effect of *Lactobacillus* as a probiotic treatment

Many attempts to treat BV with probiotics have been made, but with mixed results. Considering that *Lactobacillus* is the main genus identified in the vagina and is also the most commonly used probiotic in BV treatment, we reviewed the literature and clinical trial registrations and proposed the following three main considerations.

### Consideration 1: species variation

The first and most important issue is the *Lactobacillus* species that have been used in clinical trials. While probiotic attempts have focused on the genus *Lactobacillus*, species within a genus are not interchangeable^43^. Notably, vaginal *Lactobacillus* species are different from gut *Lactobacillus*, and their functional repertoire and ideal growing conditions markedly differ^43,44^. Species-specific characteristics may affect bacterial colonization or the microenvironment for bacteria–bacteria and bacteria–host interactions. Among the common *Lactobacillus* species that dominate the vaginal microbiome, *L. crispatus*-dominated vaginal microbiome demonstrate high stability, whereas *L. gasseri* and/or *L. iners* are more conducive to the incidence of abnormal vaginal microbiome in longitudinal analysis^46^. Moreover, even within the same species, different *L. crispatus* strains from a vaginal tract or intestinal tract demonstrate phenotypic variations that allow the bacteria to adapt to the different environments^47,48^. Furthermore, several recent studies have also indicated that different strains of vaginal *L. crispatus* demonstrate significant differences in genes for glycosylation and glycogen degradation, as well as antimicrobial and inflammatory properties, which may affect the colonization efficiency of *L. crispatus* and also the treatment effect on BV^49,50^.

Surprisingly, through extensive literature search, we found only a few BV probiotic trials regarding *L. crispatus*. Almost all of the clinical trials for BV treatment used *Lactobacillus* species from the gastrointestinal tract, probably due to how recently sequencing studies revealed that the *Lactobacillus* species dominating the vaginal microbiome are different from gut *Lactobacillus* (Tables 1–4). One *L. crispatus* clinical trial showed promising results for BV, with an 80% remission rate compared with a 59% remission rate in the placebo group^51^. This result is comparable to VMT treatment (80% vs. 80% cure rate based on clinical diagnosis), which suggests that one strain of *L. crispatus* may be as effective as VMT. Furthermore, two well-designed, double-blinded clinical trials with *L. crispatus* CTV-05 (LACTIN-V), administered to the vaginal tract directly after metronidazole treatment, showed a significantly decreased recurrence of bacterial vaginosis and increased *L. crispatus* colonization^52,53^. In the limited clinical studies described above, *L. crispatus* treatment resulted in a cure rate of 100% when considered short term and cure rates of 70% and 79.5% based on clinical Amsel’s criteria when considering long-term effects^51,53,54^.

**Table 1:** Short-term clinical trials (<4 months) using probiotics for bacterial vaginosis (BV) treatment without antibiotics.

Studies	Size	Type of study	Duration	Time	Route of administration	Probiotics and cure rate	Probiotics (CFU)	Control (Placebo)	Control (Antibiotics)	Control (Blank)	Control (Other)	Statistics (Control vs. Probiotics)
Reid et al., 2003^64^	64	R, PC	90 days	60 days	Oral capsule	*L. rhamnosus GR-1* and *L. fermentum RC-14* (37%)	>10^9^	Yes (13%)				*p* = 0.02
Hantoushzadeh et al., 2012^97^	300	R, AC	1 week	Twice a day/week	Oral yogurt	*L. bulgaris*, *Streptococcus thermophilus*, *L.acidophilus*, and *Bifidobacterium lactis* (80%)	100 g yogurt		Clindamycin (84%)			*p* = NS
Vujic et al., 2013^55^	544	R, PC, DB	12 weeks	6 weeks	Oral capsule	*L. rhamnosus GR-1* and *L. reuteri RC-14* (51.1%)	>10^9^	Yes (20.8%)				*p* < 0.001
Hallén et al., 1992^98^	57	R, DB, PC	40 days	6 days	Vaginal plug	*L. acidophilus* (21%)	10^8–9^	Yes (0%)				*p* = NS
Neri et al., 1993^99^	84	R, AC	8 weeks	7 days	Vaginal douche	*L. acidophilus* (88%)	10^8^			Yes (5%)	5% acetic acid (38%)	*p* < 0.001 *p* = 0.04
Parent et al., 1996^100^	32	R, PC	4 weeks	6 days	Vaginal tablet	*L. acidophilus* (88%)	≥10^7^	Yes (22%)				*p* < 0.05
Ozmen et al., 1998^67^	211	R, AC	1 menstrual period	12 days	Vaginal suppository	*L. acidophilus* (55.6%)	10^7^–7 × 10^8^		Metronidazole (87.7%)			*p* < 0.001
Anukam et al., 2006^56^	40	R, OB, AC	30 days	5 days	Vaginal capsule	*L. rhamnosus GR-1* and *L. reuteri RC-14* (88%)	10^9^		Metronidazole (55%)			*p* = NS
Mastromarino et al., 2009^101^	34	R, DB, PC	3 weeks	7 days	Vaginal tablet	*L. brevis CD2*, *L. salivarius FV2*, and *L. plantarum FV9* (61%)	≥10^9^	Yes (19%)				*p* < 0.05
Ya et al., 2010^102^	120	R, DB, PC	2 months	Two separate 7 days	Vaginal capsule	*L. rhamnosus*, *L. acidophilus*, and *Streptococcus thermophilus* (84.2 %)	8 × 10^9^	Yes (55%)				*p* < 0.001
Ling et al., 2013^103^	55	R, AC	30 days	10 days	Vaginal smear	*L. delbrueckii subsp. lactis DM8909* (96%)	>10^9^		Metronidazole (70%)			*p* = 0.013
Vicariotto et al., 2014^104^	34	R, PC	56 days	28 days	Vaginal tablet	*L. fermentum LF15* and *L. plantarum LP01* (83.3%)	4 × 10^8^	Yes (10%)				*p* < 0.001

Note: The studies are cited in chronological order separated by oral and vaginal administrations.

*R* randomized, *DB* double blind, *PC* placebo controlled, *OB* observer blind, *AC* active controlled, *CFU* colony-forming unit, *NS* not significant.

**Table 2:** Long term clinical trials (≥4 months) using probiotics for BV treatment without antibiotics.

Studies	Size	Type of study	Duration	Time	Route of administration	Probiotics and cure rate	Probiotics (CFU)	Control (Placebo)	Control (Blank/Other)	Statistics (Control vs. Probiotics)
Ehrström et al., 2010^105^	95	R, PC, DB	6 months	5 days	Vaginal capsule	*L. gasseri LN40*, *L. fermentum LN99*, *L. casei subsp. rhamnosus LN113*, and *P. acidilactici LN23* (48%)	>10^8^ CFU	Yes (45%)		*p* = NS
Bisanz et al., 2014^60^	14	R, DB, PC	129 days	3 days	Vaginal smear	*L. rhamnosus GR-1* and *L. reuteri RC-14* (20%)	2.5 × 10^9^	Yes (10%)		*p* = NS

Note: *R* randomized, *DB* double blind, *PC* placebo controlled, *OB* observer blind, *AC* active controlled, *CFU* colony-forming unit, *NS* not significant.

**Table 3:** Short-term clinical trials (<4 months) using probiotics for BV treatment with antibiotics.

Studies	Size	Type of study	Duration	Antibiotics treatment first	Time	Route of administration	Probiotics and cure rate	Probiotics (CFU)	Control (Placebo)	Control (Blank)	Statistics (Control vs. Probiotics)
Anukam et al., 2006b^57^	125	R, DB, PC	30 days	Oral 500 mg metronidazole for 7 days	30 days	Oral capsules	*L. rhamnosus gR-1* and *L. reuteri RC-14* (88%)	10^9^	Capsule (40%)		*p* < 0.001
Martinez et al., 2009^58^	64	R, DB, PC	28 days	Oral 2000 mg tinidazole for 28 days	28 days	Oral capsule	*L. rhamnosus GR-1* and *L. reuteri RC-14* (87.5%)	10^9^	Capsule (50 %)		*p* = 0.001
Laue et al., 2018^54^	34	R, DB, PC	38 days	Oral 500 mg metronidazole twice a day for 1 week	4 weeks	Oral yogurt	*L. crispatus LbV 88, L. gasseri LbV 150* *N, L. jensenii LbV 116*, and *L. rhamnosus LbV96* (100%)	Each 10^7^	Chemically acidified milk (64.7%)		*p* = 0.018
Ozmen et al., 1998^67^	210	R, AC	1 menstrual period	Oral metronidazole 500 mg twice daily for 1 week	12 days	Vaginal suppository	*L. acidophilus* (92.7%)	10^7^–7 × 10^8^		Yes (87.7%)	*p* *=* NS
Eriksson et al., 2005^106^	187	R, DB, PC	2 menstrual periods	Vaginal 100 mg clindamycin for 3 days	1 menstrual period	Vaginal tampons	*L. gasseri*, *L. casei var. rhamnosus*, *L. fermentum* (56%)	10^8^	Tampons (62%)		*p* = NS
Marcone et al., 2008^107^	84	R, AC	90 days	Oral Metronidazole 500 mg twice a day for 7 days	Once a week for 2 months	Vaginal tablet	*L. rhamnosus* (88%)	>4 × 10^4^		Yes (71%)	*p* = 0.05
Petricevic and Witt, 2008^108^	190	R, OB, PC	4 weeks	Oral 300 mg clindamycin for 7 days	7 days	Vaginal capsules	*L. casei rhamnosus* (83%)	10^9^	Capsule (35%)		*p* < 0.001

Note: The studies are cited in chronological order separated by oral and vaginal administrations.

*R* randomized, *DB* double blind, *PC* placebo controlled, *OB* observer blind, *AC* active controlled, *CFU* colony-forming unit, *NS* not significant.

**Table 4:** Long term clinical trials (≥4 months) using probiotics for BV treatment with antibiotics.

Studies	Size	Type of study	Duration	Antibiotics treatment first	Time	Route of administration	Probiotics and cure rate	Probiotics (CFU)	Control (Placebo)	Control (Blank)	Statistics (Control vs. Probiotics)
Hummelen et al., 2010^59^	42	R, DB, PC	6 months	Oral 400 mg metronidazole twice daily for 10 days	25 weeks	Oral capsule	*L. rhamnosus GR-1* and *L. reuteri* RC-14 (42%)	2 × 10^9^	Capsule (40%)		*p* = NS
Heczko et al., 2015^65^	154	R, DB, PC	6 months	Oral metronidazole for 7 days	10 days	Oral capsules	*L.gasseri 57* *C*, *L. fermentum 57* *A*, and *L. plantarum 57B* (54.8%)	≥10^8^	Capsule (53%)		*p* = 0.087
Russo et al., 2019^66^	48	R, DB, PC	6 months	Oral 500 mg metronidazole twice a day for 1 week	10 days per month	Oral capsule	*L. acidophilus GLA-14* and *L. rhamnosus HN001* (70.83%)	5 × 10^9^	Capsule (41.67 %)		*p* < 0.05
Larsson et al., 2008^109^	100	R, DB, PC	6 menstrual periods	Vaginal 2% clindamycin for 7 days	10 days for three menstrual cycles	Vaginal gelatine capsules	*L. gasseri Lba EB01-DSM 14869* and *L. rhamnosus Lbp PB01-DSM 14870* (65%)	10^8^–10^9^	Capsule (46%)		*p* = 0.042
Marcone et al., 2008^107^	84	R, AC	180 days	Oral Metronidazole 500 mg twice a day for 7 days	Once a week for 2 months	Vaginal tablet	*L. rhamnosus* (83%)	>4 × 10^4^		Yes (67%)	*p* = NS
Marcone et al., 2010^110^	46	R, AC	12 months	Oral 500 mg metronidazole for 7 days	6 months	Vaginal capsule	*L. rhamnosus GR-1* and *L. reuteri RC-14* (not shown)	>4 × 10^4^		Yes (not shown)	*p* = NS
Bradshaw et al., 2012^111^	268	R, DB, PC	6 months	Vaginal metronidazole for 7 days	12 days	Vaginal pessary	*L. acidophilus KS400* (72%)	≥10^9^	Pessary (73%)		*p* = NS
Recine et al., 2016^112^	250	R, AC	9 months	Oral 500 mg metronidazole twice a day for 1 week	7 months	Vaginal tablet	*L. rhamnosus* BMX 54 (79.7%)	≥10^4^		Yes (20.3%)	*p* < 0.0001
Bohbot et al., 2018^51^	78	R, DB, PC	196 days	Oral 500 mg metronidazole twice a day for 1 week	14 days	Vaginal capsule	*L. crispatus IP 174178* (79.5%)	10^9^	Capsule (59%)		*p* = 0.049
Cohen et al., 2020^62^	228	R, DB, PC	24 weeks	Vaginal 0.75% metronidazole for 5 days	10 weeks	Vaginal applicators	*L. crispatus CTV-05* (70%)	2 × 10^9^	Inactive ingredient (55%)		*p* *=* 0.01

Note: The studies are cited in chronological order, separated by oral and vaginal administrations.

*R* randomized, *DB* double blind, *PC* placebo controlled, *OB* observer blind, *AC* active controlled, *CFU* colony-forming unit, *NS* not significant.

Other *Lactobacillus* clinical trials mainly chose *Lactobacillus* species found in the gastrointestinal tract, most commonly *L. rhamnosus GR-1* and *L. reuteri RC-14*, which yielded a cure rate of between 51% and 88% when considered short term (<4 months)^55–58^ (Tables 1 and 3) and between 20% and 42% when considered long term (≥4 months)^59,60^ (Tables 2 and 4). Improper *Lactobacillus* species may partly explain why the cure rate varies from study to study and why the bacteria do not colonize the vaginal tract. Notably, *L. crispatus* is strongly associated with a reduced risk of BV compared with other *Lactobacillus* species (Tables 3 and 4). Interestingly, even the placebo branch of the clinical trials showed a large range of cure rates (0–73%; Table 1), suggesting the complicated dynamics of BV and its treatment. Another possible reason might be the subjectivity of the diagnostic methods, which used wet mounts and Gram staining. A more accurate evaluation of treatment effects, such as sequencing, should be considered in future clinical trials^7,8^. In addition to *L. crispatus* CTV-05 (LACTIN-V), several other *L. crispatus* clinical trials aimed at preventing recurrent urinary tract infection also demonstrated safe and efficient use in the vaginal tract^61–63^.

To summarize, most of the current BV clinical trials did not use *Lactobacillus* species from the vaginal tract. These data collectively indicate that *L. crispatus* could be of potential use for BV treatment and that a rigorous pre-clinical screening strategy needs to be applied to identify the best strains that can maximize adaptiveness and colonization in the vaginal environment. The proper *Lactobacillus* species from a vaginal microbiome should also be tested in large, randomized, placebo-controlled cohorts.

### Consideration 2: administration method

The second important factor that we believe contributes to the inefficient cure rate in clinical trials is the mode of probiotic administration. Although there is evidence that the gut microbiome might influence the vaginal environment, oral intake of bacteria for vaginosis treatment is probably based on the immune response or circulating metabolites that lack direct bacteria–bacteria inhibition^64^. Oral administration of probiotics follows the regulation of food supplements instead of drug development, which is less strict and provides a faster track to the market. The ability of probiotic strains to survive passage through the gastrointestinal tract becomes an important selection criterion when oral administration is intended^54,59,65,66^. Hypothetically, vaginal administration allows for the direct replacement of BV-related microbes by probiotic strains. Once these strains have colonized, the replacement consequently results in the maintenance of a low pH and the production of lactic acid and antimicrobial substances, which could further support a healthy vaginal microbiome environment on site^16^. Direct vaginal application also showed a slightly higher cure rate compared to the same *L. rhamnosus* GR-1 and *L. reuteri* RC-14 strains administered orally (88% vs. 51%; Table 1)^55,56^.

### Consideration 3: pretreatment

Finally, the vaginal microenvironment is altered by BV-related bacteria, which could increase the difficulty for probiotic strains to compete with BV-related bacteria and hinder the colonization of probiotic strains^52^. Thus, it may be necessary to open a niche for probiotic strains to minimize colonization resistance from resident bacteria, especially overgrown biofilm-forming bacteria. Combinations of antibiotics and probiotic treatments have been previously attempted. A study indicated that the combination of probiotics and metronidazole is more effective than antibiotics alone in maintaining a healthy vaginal ecosystem^67^. There is also an overall higher remission rate with clinical trials with combined probiotic and antibiotic treatment (42–83%) compared to those using probiotics alone (20–48%) in long-term studies (≥4 months) (Table 4 vs. Table 2). For instance, short-term studies (<4 months) on *L. rhamnosus GR-1* and *L. reuteri RC-14* showed an 88% cure rate with antibiotic pretreatment compared to 51–88% in *L. rhamnosus GR-1* and *L. reuteri RC-14* only without antibiotic pretreatment (Tables 1 and 3)^55,56,58^. Moreover, long-term studies (≥4 months) on *L. rhamnosus GR-1* and *L. reuteri RC-14* showed a 42% cure rate with antibiotic pretreatment compared to 20% on *L. rhamnosus GR-1* and *L. reuteri RC-14* only without antibiotic pretreatment^59,60^ (Tables 2 and 4). Given that orally administered antibiotics influence the whole gut microbiome^36,37^, we propose larger randomized cohort studies with *L. crispatus* delivered directly to the vagina after pretreatment with antibiotics administered vaginally that reduce the influence of BV-related bacteria (Fig. 1).

### VMT

VMT uses a similar approach as fecal microbiome transplant (FMT) which has greatly developed in the past decade in the field of gastroenterology, most prominently to treat recurring *Clostridioides difficile* infections^12^. VMT is the process of obtaining vaginal fluid from a donor and administer it into the vagina of a recipient, after thorough testing and minimal processing with the goal of maintaining the viability of the bacteria^68^. The mixture of fluid not only includes the microbes from the donor but also potentially cells, bacteriophages, proteins such as cytokines, and metabolites such as lipids and antimicrobial peptides. Recently, a study recruited five patients suffering from recurrent BV and introduced treatment with VMT after an antibiotic regimen^69,70^. Four of five patients had long-term remission after VMT, making it a promising alternative treatment for recurrent BV. Further studies including large, randomized, placebo controlled clinical trials are needed to follow up on VMT. Notably, of the five women included in the VMT study, four became colonized by *L. crispatus* with a full cure and were symptom-free up to 11 months, although three of the women required three rounds of VMT before achieving sustained remission. A fifth woman was colonized by *L. gasseri* and had only a partial cure based on clinical criteria. The feasibility of transplanting the vaginal microbiome between women and its protection against BV development is further supported by increasing evidence from women who have sex with women. The interchange of the vaginal microbiome during sex leads to a high level of concordance for a stable vaginal microbiome and a low risk of BV^69,71^.

Overall, VMT presents a promising way to combine antibiotic treatment and restoration of the vaginal microbiome to combat vaginosis-related bacteria. It also provides a whole environment, including the mixture of vaginal microbes and molecules produced by both hosts and microbes (e.g., lactic acid, cytokines, bacteriocins, and antimicrobial peptides), which assists in the colonization of essential bacteria while working against BV-associated bacteria^9,11,14^. These molecules might be essential for the successful re-establishment of a healthy vaginal microbiome.

However, the main functional compounds of VMT have yet to be identified. Further, similar to FMT, attention has been drawn to potential risks including heterogeneity across donors and the transmission of infectious agents and metabolites outside the standard set of tests^72,73^. Also, due to the lack of a standardized manufacturing process in terms of the definition of microorganisms, dose, functional properties, antibiotic resistance profiling, and potential presence of pathogens or contaminating microorganisms, it is still challenging to overcome these obstacles and pass the standard from federal agencies such as FDA. Other ethical issues, including the ethnicity and socioeconomic status of women, also need to be considered before VMT. The FDA issued a special guidance for FMT and recently drafted a guidance for developing drugs for BV treatment^74^. However, since BV recurrence is not as deadly as *C. difficile* infections, whether VMT benefits outweigh risks remains an open question. Moreover, VMT is still in its infancy, lacking large clinical trial data, and whether VMT provides a better clinical cure rate than defined probiotics and/or prebiotics needs further investigation. Further medical and regulatory needs for the clinical and regulatory viability of VMT include standardized procedures for donor screening, laboratory tests to exclude potential risk of infection, standardized sample preparation and administration procedures, standardized protocols for follow-up of donors and recipients, and maintenance of records in a biobank as currently proposed for FMT^69,75,76^.

### Other possibilities

Other possibilities that could replace antibiotics as vaginosis treatment or pretreatment should also be evaluated. Isolating bacteriophages is a well-established technology, and bacteriophages targeting BV-related bacteria can be used alone or in combination with probiotics^77^. Previous studies have identified that *Lactobacillus* bacteriophages are related to BV, and a higher load of *Lactobacillus* bacteriophages was found in vaginal microbiome samples among women with BV compared to healthy women^78,79^. However, although there have been bacteriophages against *Gardnerella* and *Clostridium* reported by sequencing, no lytic bacteriophage has been isolated by culturing^80^. Furthermore, bacteriophages targeting *Prevotella* have been reported in the gut, but whether bacteriophages targeting vaginal *Prevotella* and other BV-related bacteria exist should be further studied^80^.

One aspect of the high rate of BV recurrence after therapy could be due to biofilm persistence^81^. Biofilm formation enhances the endurance of BV-related bacteria against antibacterial regimens from beneficial vaginal microbes or antibiotic treatment^82^. *G. vaginalis* is considered to be the key player in biofilm formation by adhering to the surface of vaginal epithelial cells and allowing the attachment of other species, thus leading to the formation of “clue cells,” which have been used in the clinical diagnosis of BV^83,84^. Bioproducts, such as anti-biofilm or antimicrobial peptides that inhibit BV-associated bacterial growth and biofilm formation, could be a future replacement for antibiotic treatment to achieve higher precision and fewer side effects^31,85,86^. Biofilm-disrupting agents, such as intravaginal boric acid enhanced with ethylenediaminetetraacetic acid (TOL-463) and amphoteric tenside (WO3191), are being investigated to determine their role in BV treatment (NCT03930745, NCT02687789)^87,88^. Another biofilm-disrupting agent example is a pHyph, a vaginal pessary containing glucono-delta-lactone and sodium gluconate. In a recent study, it was shown that pHyph has the potential to restore a normal pH and resolve clinical BV symptoms^89^.

*Lactobacillus* monoisolate or mixtures of healthy vaginal bacterial strains, with combinations of beneficial molecules, could be additional options for treatment^72^. Another promising approach currently in clinical trial is the Flourish Vaginal Care System (ClinicalTrials.gov, Identifier: NCT03734523)^90^, which includes bio-matched vaginal secretions of women with *L. crispatus*-dominated microbiome, a probiotic combination of *L. crispatus* and other strains, and a gentle, pH-balancing cleanser. All of these methods including a probiotic mix, prebiotic combinations, and/or bacteriophages provide more controlled conditions, convenience for clinical application, and ease of commercialization compared to VMT.

### Challenges/opportunity of treating BV using novel interventions including probiotics and VMT

The golden age for restoring the vaginal microbiome to decrease BV and its recurrence has begun. However, except for the disadvantages discussed above, several other challenges need to be considered. Foremost, unlike regular drugs, VMT and probiotics lack a standardized manufacturing process, which could affect microbial survival, growth, and viability^45,91,92^. A standardized procedure for producing VMT and probiotics should be established and tested. Manufacturing the whole vaginal microbiome consistently and stably in vitro will contribute to the development and approval processes for the clinical use of VMT. Also, the effects of probiotics are strain-specific and dose-dependent; hence, medical-grade probiotics require certified laboratories universally shared validated and standardized methodologies for production and quality-control^45^.

Second, suitable regulatory aspects related to the production and marketing of VMT and vaginal probiotics should be in place. Vaginal administration leads to products not classified as dietary supplements. Being classified into personal care products or being prescribed to patients as drugs needs more restrictive regulation and report adverse events^91,93^. In this regard, professional medical associations should issue recommendations concerning the role of VMT and probiotics in obstetrics and gynecology, as their uncontrolled implementation might also lead to a potential decrease in effectiveness. Detailed discussions on medical and regulatory considerations, including finding the right FDA regulatory path for VMT, are of crucial importance for future clinical trials of VMT and have been reviewed in other papers^69,94^.

Finally, further high-quality data are needed to define the microbiome/strains and their effective dose in different obstetrical and gynecological conditions. Furthermore, more research needs to be focused on the interactions between vaginal microbes^95^, including pathogens and potential probiotics^96^, as well as between host and microbes^84^. In this manner, suitable probiotics can be selected for patients with different disease conditions or other background characteristics. Finally, vaginal microbe biobanks, such as biobanks of different *L. crispatus* strains, should be built, sequenced, and well documented so that more probiotics or probiotic cocktails can be selected and tested^50,92^.

## Conclusion

In summary, we provide an overview of current treatments and interventions for BV, and discuss their advantages and limitations. We propose possible reasons why some recent clinical trials using probiotics did not work as efficiently as expected. We believe the current high recurrence rate of BV is mainly due to the application of microbial species that do not originate from the vagina, an oral instead of vaginal administration method, and a lack of probiotic replacement after antibiotic treatment. We believe there is a great opportunity to use vaginal *Lactobacillus* species such as *L. crispatus*, instead of gut *Lactobacillus* species as in earlier clinical trials, administered directly into the genital tract in combination with pretreatments such as vaginal antibiotic treatment, anti-biofilm, or antimicrobial agents for BV treatment (Fig. 1). There is a great need for large, placebo controlled, double blind clinical trials and mechanism-based research to determine the safety and efficacy of these novel interventions. The dynamic and complex vaginal microbiome creates obstacles for clinical trials, and the considerations discussed here should help accelerate the successful development of clinical trials against BV.

## Data Availability

All data generated in this study are included in this published article.
